# Advances in Periodontal Pathogens

**DOI:** 10.3390/microorganisms10071439

**Published:** 2022-07-16

**Authors:** Irina-Georgeta Sufaru, Maria-Alexandra Martu, Sorina Mihaela Solomon

**Affiliations:** Department of Periodontology, Grigore T. Popa University of Medicine and Pharmacy, Universitatii Street 16, 700115 Iasi, Romania; ursarescu.irina@umfiasi.ro (I.-G.S.); sorina.solomon@umfiasi.ro (S.M.S.)

Even though periodontitis is considered an infectious disease, there are a number of factors that distinguish it from other infectious diseases: it is not the result of infection with an individual pathogen, but rather the consequence of a modified microbial community interaction with the host organism. Thus, periodontitis becomes a dysbiotic disease, which translates into alterations of certain pathogens concentrations or their influence in the subgingival biofilm that will trigger and maintain a periodontal inflammatory status [1].

The immune response of the host to dysbiosis phenomena is one of an inflammatory nature; in certain conditions, this response can become hyperinflammatory, further favoring the dysbiotic status. In addition, the periodontal pocket, through the low oxygen concentration and the protection of the gingival soft wall [2], creates an extremely favorable environment for the development of the subgingival biofilm. Moreover, a number of genetic and environmental factors can mark the interaction between periodontal pathogens and the host, aggravating the inflammatory status and accelerating the destruction of periodontal tissues. It is essential to correctly identify the component causes, in order to properly conduct preventive and curative measures. Even if microbial “invasion” was detected in periodontal lesion biopsies, it is not certain that this process is evidence for infection or for the role in periodontitis evolution [3]; moreover, it was demonstrated that putative pathogens can trigger inflammation without invading periodontal tissues [4].

Studies based on bacterial culture have shown that the composition of subgingival bacterial communities varies in different periodontal statuses—health, gingivitis and periodontitis [5]. Of course, the development of investigative and laboratory technologies has facilitated the characterization of the oral microbiome with high taxonomic resolution, clearly demonstrating that there are unique microbial signatures for each mentioned state [6].

*Porphyromonas gingivalis* has been called a “keystone pathogen”, meaning “a key species that supports and shapes a microbial community in ways that also promote the pathogenesis of the disease.” [7]; thus, according to this particular definition, *P. gingivalis*, even in low abundance, can orchestrate inflammatory disease by remodeling a normally benign microbiota into a dysbiotic one [8]. Therefore, even in low concentrations, key pathogens can disrupt the host’s immune response, triggering destructive tissue phenomena. With the evolution of periodontitis, the concentration of these pathogens can increase, becoming dominant, accelerating the processes of synergy and dysbiosis [9]. The bacterial biofilm functions as a community, on the model of a “megalopolis”, where the survival and functionality of pathogens with increased virulence depend on other members of the community, just as the dominant species can influence the existence of other species, contributing together to tissue homeostasis disruption.

Metagenomic and metatranscriptome analyzes were performed in dynamics, at successive visits, to characterize the bacterial profile and the signature of the molecular activity associated with the progression of active periodontitis [10]. Sites were divided into active sites (periodontal clinical attachment loss greater than 2 mm from baseline) and inactive sites (differences in measurement of attachment loss less than 1 mm). The authors reported that there were no significant changes in the bacterial community in the stable sites. At sites with active tissue loss, several *Streptococcus* spp. significantly dominated the microbial profile at baseline, while several bacteria of the genus *Prevotella* and *Synergistetes* were present at sites that showed disease progression at follow-up visits. Moreover, the authors observed that bacteria that are not conventionally associated with periodontal disease, such as the genus *Streptococcus*, *Vellonella parvula* and *Pseudomonas fluorenscens*, produced large amounts of virulence factors [10].

A consequence of these findings is the emergence of the term “nosoymbiocity”, which describes the collective pathogenic potential of the microbial community [11], in which the absolute dichotomous notions of “pathogen” and “commensal” lose their relevance. A variety of factors can promote the transition from eubiosis to dysbiosis. Factors influencing the host’s immune-inflammatory response may alter the composition or metatranscriptional image of a polymicrobial community or the ability of certain bacteria to translocate to normally sterile sites [11,12].

It is already universally accepted that periodontal disease does not have a linear evolution and, moreover, can be extremely heterogeneous from one individual to another or even during the life of the same individual, under the influence of environmental factors, aging, epigenetic phenomena or the appearance of certain pathological changes (cardiovascular disease, diabetes, etc.). Thus, the therapeutic challenge lies precisely in the correct identification of the factors that led to the appearance and evolution of periodontitis, in order to establish an appropriate multidisciplinary treatment that includes all potential factors. However, the microbial community remains an important component in the etiopathogenic picture of periodontitis and the understanding of microbial interactions in the biofilm in dynamics could generate new therapeutic principles.

Even though scaling and root planning are the “gold standard” in periodontal therapy, several discoveries have led to the use of various adjunct physical and/or chemical methods of periodontal treatment. However, therapeutic guidelines recommend caution in their use, either due to side effects that cannot be ignored (such as for antibiotics, bisphosphonates or anti-inflammatory drugs) or due to the scarcity of available data in the literature, insufficient to promote a stable and secure protocol.

Recent findings on the interactions between the species that populate the supra- and sub-gingival biofilm, as well as the influence of dysbiosis and various local and systemic factors in the occurrence and evolution of periodontitis will be presented in this special issue. Moreover, future research on different forms of periodontal therapy will be presented by invited leading authors and our research group.
